# Secretion of Proteases by an Opportunistic Fungal Pathogen *Scedosporium aurantiacum*

**DOI:** 10.1371/journal.pone.0169403

**Published:** 2017-01-06

**Authors:** Zhiping Han, Liisa Kautto, Helena Nevalainen

**Affiliations:** 1 Department of Chemistry and Biomolecular Sciences, Macquarie University, Sydney, Australia; 2 Biomolecular Frontiers Research Centre, Macquarie University, Sydney, Australia; Uniwersytet Gdanski, POLAND

## Abstract

*Scedosporium aurantiacum* is an opportunistic filamentous fungus increasingly isolated from the sputum of cystic fibrosis patients, and is especially prevalent in Australia. At the moment, very little is known about the infection mechanism of this fungus. Secreted proteases have been shown to contribute to fungal virulence in several studies with other fungi. Here we have compared the profiles of proteases secreted by a clinical isolate *Scedosporium aurantiacum* (WM 06.482) and an environmental strain (WM 10.136) grown on a synthetic cystic fibrosis sputum medium supplemented with casein or mucin. Protease activity was assessed using class-specific substrates and inhibitors. Subtilisin-like and trypsin-like serine protease activity was detected in all cultures. The greatest difference in the secretion of proteases between the two strains occurred in mucin-supplemented medium, where the activities of the elastase-like, trypsin-like and aspartic proteases were, overall, 2.5–75 fold higher in the clinical strain compared to the environmental strain. Proteases secreted by the two strains in the mucin-supplemented medium were further analyzed by mass spectrometry. Six homologs of fungal proteases were identified from the clinical strain and five from the environmental strain. Of these, three were common for both strains including a subtilisin peptidase, a putative leucine aminopeptidase and a PA-SaNapH-like protease. Trypsin-like protease was identified by mass spectrometry only in the clinical isolate even though trypsin-like activity was present in all cultures. In contrast, high elastase-like activity was measured in the culture supernatant of the clinical strain but could not be identified by mass spectrometry searching against other fungi in the NCBI database. Future availability of an annotated genome will help finalise identification of the *S*. *aurantiacum* proteases.

## Introduction

Members of *Scedosporium* spp. are ubiquitous in nature and can be isolated from a wide range of human-impacted environments [1, 2]. Some species of the *Scedosporium* spp. complex such as the recently identified *S*. *aurantiacum* are opportunistic pathogens that affect people with diabetes, solid tumours, chronic lung diseases and stem cell transplants [3]. In Australia, *S*. *aurantiacum* is the second most common filamentous fungus isolated from the sputum of cystic fibrosis (CF) patients after *Aspergillus fumigatus* [4, 5].

The majority of the work carried out with *S*. *aurantiacum*, currently described in the literature, features prevalence studies [4, 6, 7], animal studies including virulence [8–10] and antifungal susceptibility studies [1, 11, 12]. Recently, we have reported phenotypic profiling of this fungus that revealed differences in the carbon substrate utilisation patterns between clinical and environmental *S*. *aurantiacum* isolates [13].

A correlation between production of proteases and fungal pathogenicity to humans has been established in the studies with pathogenic *Aspergillus* spp. [14–16], *Candida* spp. [17, 18] and Dermatophytes [19]. Proteolytic activities identified from these fungi feature the families/subfamilies of elastase-like, chymotrypsin-like, subtilisin-like, and trypsin-like serine proteases, aspartic proteases, metalloproteases and cysteine proteases [16, 20, 21]. Amongst the proteases, elastase activity has emerged as the main indication for virulence and has been linked to germination and penetration into mice lungs [22], deterioration of respiratory function [14], and lung injury [23]. Secreted serine proteases and cysteine proteases from *A*. *fumigatus* strain AF293 were able to breach the alveolar epithelial cell barrier by disruption of the actin cytoskeleton and sites of focal adhesion in human lung cancer cells [16]. Extracellular aspartic proteases have been implicated in the virulence of *C*. *albicans* in a mouse model; these proteases were found to have broad substrate specificity, degrade many mouse proteins and digest cells and molecules of the host immune system [24].

Secreted proteases may also contribute to the virulence of the fungal species of the *Pseudoallescheria boydii* complex including *S*. *aurantiacum* [6, 25, 26] since research has demonstrated that infections caused by *P*. *boydii* highly resemble those of *A*. *fumigatus* [27, 28], whose secreted proteases have been reported as putative virulence factors [16, 23, 29, 30]. The first report on the proteases secreted by *P*. *boydii* was by Larcher *et al*. in 1996, who purified and characterized a 33 kDa subtilisin-like protease from the fungal culture supernatant [31]. Recently, a zinc-metalloprotease active in an acidic pH was detected in *P*. *boydii* mycelia [32]. In addition, *Scedosporium apiospermum*, a member of the *P*. *boydii* complex, was found to produce six distinct mycelial metalloproteases ranging from 28 to 90 kDa in size [33]. To date, there are no reports relating these proteases to virulence.

In this study, we have explored, for the first time, proteases secreted by *S*. *aurantiacum*, as a prelude to studies into potential roles of these proteases in fungal virulence. Protease profiles were compared between a clinical isolate and an environmental strain. The amount, type, and activity of major proteases secreted by these two strains were determined in response to cultivation on synthetic cystic fibrosis sputum medium supplemented with mucin or casein.

## Materials and Methods

### Liquid cultivation media

Synthetic cystic fibrosis sputum medium (SCFM) containing mineral salts, amino acids and 1% (w/v) glucose was used as a base for all liquid media and prepared as previously described [34]. Additions to SCFM featured 1% (w/v) casein from bovine milk (Sigma-Aldrich, Australia; SCFM+C) or 1% (w/v) mucin from porcine stomach (type III; Sigma-Aldrich, Australia; SCFM+M). Mucin and casein were suspended in Milli-Q H_2_O; sodium hydroxide was added to dissolve casein. These substrates were autoclaved separately and mixed with SCFM just before use. SCFM was sterilized by filtering through a 0.22 μm membrane (Millipore) and final pH of media was adjusted to 5.7 to support fungal growth [35].

### Fungal strains and cultivation conditions

The *Scedosporium aurantiacum* strains studied were WM 06.482 (clinical isolate) isolated from broncho-alveolar lavage of a CF patient in Australia and WM 10.136 (INS1120; environmental strain) originating from a valley near Innsbruck, Austria [8]. The strains were obtained from the culture collection of the Medical Mycology Laboratory, Centre for Infectious Diseases and Microbiology, Westmead Hospital, Sydney, Australia. Virulence status of WM 06.482 has been established using immunocompromised mice [8]. In this paper, no significant virulence differences were found between WM 06.482 and environmental strains examined. The virulence of both strains used in the current study has been assessed using the invertebrate wax moth *Galleria mellonella* larval model, and results showed that the clinical isolate WM 06.482 was about three times more virulent than the environmental strain WM 10.136 [13]. However, there are no published reports regarding comparison of these strains in a mammalian model to establish their relative virulence.

*S*. *aurantiacum* strains were cultured on Malt Extract agar (Oxoid, Australia) at 37°C for 7 to 10 days to attain sufficient conidiation. Conidia were harvested by gentle agitation into a solution containing 0.9% (w/v) sodium chloride and 0.01% (v/v) Tween 80, filtered through an autoclaved 5 mL tip packed with cotton wool to remove hyphal fragments and counted using a Neubauer haemocytometer. Liquid cultures were performed in 250 ml conical flasks containing 50 ml of growth medium, inoculated with 2×10^6^ conidia/ml and incubated for up to seven days at 37°C on an orbital shaker at 200 rpm with three individual flasks dedicated for each time point. Culture supernatants were collected at every 24 h from flasks dedicated to each time point. Contents were centrifuged at 4500 g for 30 min, and the supernatant filtered through a 0.22 μm membrane (Millipore, Australia) at 4°C. Cleared supernatants were then aliquoted in 1.5 ml Eppendorf tubes and stored at -80°C. Fungal protease inhibitor cocktail (0.05% v/v, Sigma-Aldrich, Australia) was added in the culture supernatant samples used for proteomic analysis [36].

### Measurement of growth and pH

Measurements were performed every 24 hours (three replicates). After removal of the culture supernatant, mycelia left in the centrifuge tubes were washed three times with 50 ml Milli-Q water by inverting. The washed mycelia were frozen at -30°C for at least 4 h, and then freeze dried. Biomass was calculated as the difference between the weight of the centrifuge tubes with and without the freeze dried mycelia. The starting biomass was recorded as the weight of the same amount of freeze dried conidia used to inoculate each flask. The pH of each culture supernatant was measured at room temperature.

### Protease activity assays

All protease activity assays were performed under sub-saturating conditions. General protease activity in thawed supernatant samples was assayed using azocasein (Sigma-Aldrich, Australia) as a substrate following the method of Rauscher [37] with some adjustments. Briefly, the substrate (10 mg/ml) was dissolved in citrate-phosphate buffer pH 7.5 which was deemed optimal for the assay of general protease activity (data not shown). A 75 μl aliquot of the supernatant was mixed with 50 μl substrate solution in a 96-well plate and incubated for 60 min at 37°C. The reaction was terminated by adding 125 μl 5% (w/v) trichloroacetic acid (TCA). After centrifugation at 4000 g for 5 min, the supernatant was collected and absorbance of the released azo-dye read at 366 nm and activity calculated according to the method of Coelho *et al* [38]. Protease activity is calculated as μg of azocasein digested in one minute, normalized against dry fungal biomass. Reaction blanks were prepared by adding TCA to the culture supernatant to denature proteases before addition of azocasein. General protease activity was measured every 24 hours over seven days.

The types of proteases in the culture supernatant were studied using the following class-specific inhibitors: 1 mM PMSF (serine protease inhibitor; Sigma, Australia), 5 mM EDTA (metalloprotease inhibitor; Sigma, Australia), 100 μM pepstatin A (aspartic protease inhibitor; Merck, Nottingham, UK), and 5 μM E-64 (cysteine protease inhibitor; Sigma, Australia). The day-4 culture supernatants were pre-incubated with a protease inhibitor for 30 min at room temperature prior to performing a general protease activity assay using azocasein as substrate. Culture supernatants with no inhibitor added were also included in the assay for comparison.

Based on the inhibitor studies, serine proteases (chymotrypsin, subtilisin-like, elastase-like and trypsin-like proteases), metallo and aspartyl proteases were studied in more detail using subclass specific substrates. These substrates were synthetic peptides coupled with either 7-Amino-4-methylcoumarin (MCA) or ρ-nitroanilide (ρ-NA) (Peptide Institute Inc, Japan), and protease activities were calculated based on fluorometric or colorimetric detection. Cysteine protease activity was also included in the assay. Substrates, buffers and proteins used as a positive control are listed in Table 1. Chymotrypsin-like, elastase-like, subtilisin-like, trypsin-like, cysteine, collagenase and aspartic protease activities were measured in their optimal pH described in the literature following the manufacturer’s instructions with some modifications. The reaction mixture contained 50 μl of culture supernatant and 0.5 mM substrate in 200 μl of appropriate buffer. After incubation at 37°C for 4 min, the absorbance of released ρ-NA was read at 410 nm, or fluorescence of the released MCA was measured at λex = 380 nm and λem = 460 nm at 37°C. The amount of released ρ-NA or MCA was calculated using a standard curve prepared with appropriate dilutions of ρ-NA or MCA. Activities are given as nM of MCA or μM of ρ-NA released per minute per mg of dry biomass. All assays were performed in triplicate, and statistical analysis of the biological replicates was conducted using Excel or OriginPro 8.5 (www.originlab.com).

**Table 1:** Class-specific substrates used to detect the protease class and activity in fungal culture supernatants.

Specificity	Substrate	Positive control	Buffer and assay pH	References
Chymotrypsin-like	N-Benzoyl-L-Tyrosine-ρ-NA	Bovine pancreas alpha-chymotrypsin-like	0.1 mM Tris-HCl buffer, pH 8.0	[39]
Subtilisin-like	Z-Ala-Ala-Leu- ρ-NA	Sublitisin A from *Bacillus* sp	0.1 mM Tris-HCl buffer, pH 8.0	[40]
Elastase-like	N-Sue-Ala-Ala-Ala-MCA	Elastase-like type I from porcine pancreas	0.1 mM Tris-HCl buffer, pH 8.0	[41]
Trypsin-like	Boc-Phe-Ser-Arg-MCA	Trypsin-like from bovine pancreas	67 mM sodium phosphate buffer, pH 7.6	[42]
Cysteine	Z-Arg-Arg-MCA	Papain from *Papaya latex*	100 mM sodium acetate buffer containing 10 mM DTT, pH 5.5	[43]
Collagenase	Sue-Gly-Pro-Leu-Gly-Pro-MCA	Collagenase from *Clostridium histolyticum*	50 mM tricine buffer pH 7.5 with 10 mM CaCl_2_ and 400 mM NaCl, pH 7.5	[44]
Aspartic	Boc-Leu-Ser-Thr-Arg-MCA	Pepsin from porcine gastric mucosa	0.2 M acetate buffer, pH 3.5	[45]

### Protein electrophoresis and enzyme activity zymograms

Culture supernatants were analyzed by protein electrophoresis according Laemmli [46]. An aliquot of 21 μl of supernatant from each culture was mixed with 4× NuPAGE LDS sample buffer (Thermo Fisher Scientific), heated at 70°C for 10 min, and subjected to electrophoresis at 120 V for approximately 1 h on a 12.5% (w/v) tris-glycine gel using a running buffer containing 1.4% (w/v) glycine, 0.1% (w/v) SDS and 24 mM Tris. The protein gel was stained overnight in Coomassie Brilliant Blue G-250 (Bio-Rad, CA, USA) and destained in 1% (v/v) acetic acid. To detect protease activity of the secreted proteins, another set of supernatants (not heated) was applied to zymogram gels made of 12.5% (w/v) Tris-glycine gels containing 0.1% (w/v) casein (from bovine milk, Sigma, Australia). Following electrophoresis, zymogram gels were soaked in 2.5% (v/v) Triton-X 100 to for 30 min twice and washed 3 × 10 min in MilliQ water to renaturate proteins and restore enzyme activity. The gels were then incubated for 16 h in 0.03 mol l^-1^ Tris-HCl buffer pH 7.5 at 37°C. After incubation, the zymogram gels were subjected to the same staining and destaining procedures as the SDS-PAGE gels [47].

### Sample preparation for proteomic analysis

Proteins in the day-4 culture supernatant were precipitated following the method of Fragner [48] with some modifications. Briefly, culture supernatants with protease inhibitors added were frozen and stored at -80°C for 12 h. After thawing at 4°C the samples were centrifuged at 10000 g for 1 h at 4°C and the supernatant was removed into a new tube. Na-deoxycholate (2% w/v in dd H_2_O) was added into the supernatant to the final concentration of 0.02% (w/v) and the mixture was incubated on ice for 30 min after vortex briefly [49]. Na-deoxycholate was used to improve protein recovery [50]. Then, 10% (w/v) TCA was added to the solution and the content was incubated on ice for at least 12 h. After centrifugation at 4000 g and 4°C for 5 min, the supernatant was discarded carefully and the pellet was washed twice using cold acetone (-30°C). Protein pellets were kept at -80°C until use.

### Identification of proteases

Protein pellets from above were resuspended in 50 μl Tris-buffer (50 mM, pH 8.0) and protein concentration was measured according to Bradford [51]. Twenty micrograms of protein from each sample was heated for 10 min at 70°C and subjected to electrophoresis, as described above. Each lane on the protein gel was cut across horizontally into 10 slices. Each slice was chopped finely, destained with 50% acetonitrile (ACN) in 100 mM ammonium bicarbonate, reduced with 10 mM dithiothreitol, alkylated with 55 mM iodoacetamide, and then washed twice with 25 mM ammonium bicarbonate. Gel slices were dehydrated with ACN before drying by vacuum centrifugation. The dry samples were kept on ice for 10 min and then digested with trypsin (Sequencing grade modified, Promega, Australia) using a trypsin: protein ratio of 1:30 (w/w) as described by Grinyer [52]. Biological triplicates were analyzed in parallel. In addition, GluC digestion (Sequencing Grade, Promega, Australia) was carried out in a similar fashion with a GluC: protein ratio of 1:50 (w/w) separately to the trypsin digestion. Peptides were extracted with 65% (v/v) ACN containing 1.65% (v/v) formic acid. Extracted peptides were desalted and concentrated using C18 zip-tips (Millipore, Australia), and 10 μl of the resulting peptides was subjected to reversed-phase nanoscale liquid chromatography coupled to tandem mass spectrometry (nanoLC−MS/MS) using an LTQ-XL ion-trap mass spectrometer (Thermo, San Jose, CA). In brief, peptides were injected onto an in-house packed column with Halo C18 (Advanced Materials Techology, USA) and washed with buffer A (5% (v/v) ACN containing 0.1% (v/v) formic acid). The bound peptides were then eluted from the column using a linear solvent gradient buffer B (99.9% (v/v) ACN, 0.1% (v/v) formic acid), with the following steps: 1–50% of buffer B for 58 min, 50–85% of buffer B for 2 min, held at 85% for 8 min with a flow rate of 500 nl min^-1^ across the gradient. The resolved peptides were directed into the nanospray ionization source of the mass spectrometer and scanned in the spectral range 350−2000 amu. Automated peak recognition, dynamic exclusion window set to 90s and tandem MS of the top ten most intense precursor ions at 35% normalization collision energy was performed using Xcalibur software (version 2.06, Thermo, San Jose, CA) [53].

Protein identification was carried out using the Global Proteome Machine software of the X!Tandem algorithm (GPM, http://www.thegpm.org). For each experiment, the 10 fractions were processed sequentially with output files for each individual fraction and also a merged, non-redundant output file was generated for protein identifications with log (e) values less than -1. An in-house database was created from NCBI protein entries comprising protease sequences of fungal species including *Saccharomyces* spp., *Schizosaccharomyces* spp., *Trichoderma* spp., *Neurospora* spp., *Aspergillus* spp., *Candida* spp. and *Pseudallescheria* spp. GPM search parameters included MS tolerance of ±2 Da, MS/MS tolerance of ±0.2 Da, carbamidomethylation of cysteine as fixed modifications, oxidation of methionine as variable modifications, and tolerance of two missed tryptic cleavages and K/R-P cleavages. Only the proteins that were identified in three biological replicates and had a total spectral count of at least 5 were considered as a valid protein identity [54].

## Results

### Growth and secretion of proteases in different media

**Fig 1:**
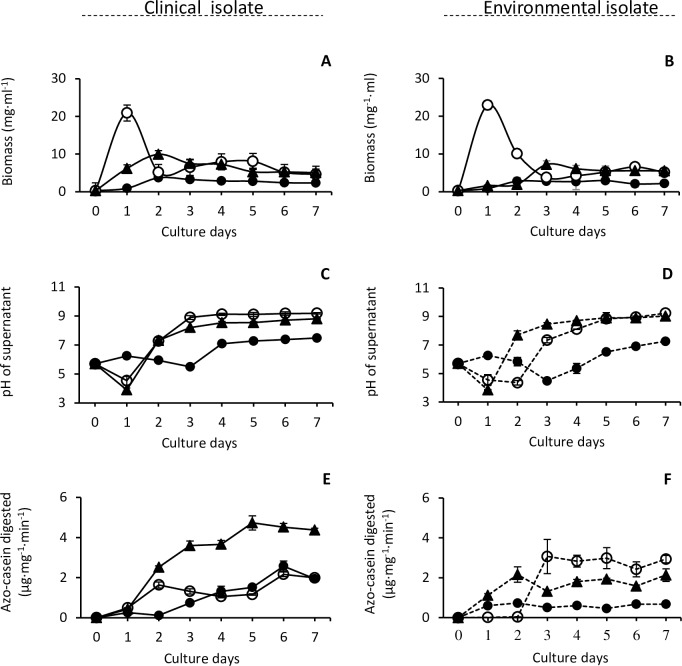
Growth and protease activity of *S*. *aurantiacum* WM 06.482 (clinical isolate) and WM 10.136 (environmental isolate) in different media. (A, B) Change in the dry weight of mycelia over time in 50 ml cultures. The data of day 0 represent the dry weight of the same amount of freeze dried conidia used to inoculate each flask. Due to the precipitating casein on day one in the casein supplemented medium, the dry weight measured encompasses both dry mycelia and casein; (C, D) pH of the culture supernatant; (E, F) general secreted protease activity, normalized against dry biomass. Data represent mean ± SD (3 biological replicates). ● SCFM: Synthetic CF sputum medium, ○ SCFM+C (casein added); ▲ SCFM+M (mucin added).

The clinical isolate *S*. *aurantiacum* WM 06.482 and the environmental strain WM 10.136 were cultured in the SCFM medium with addition of casein or mucin to boost growth and induce protease production. The growth rates were investigated by measuring the dry weight of the biomass from day zero to day seven. As shown in Fig 1A and 1B, the clinical and environmental strains showed a similar overall growth pattern in all media with some exceptions. The clinical isolate seemed to grow faster on SCFM supplemented with mucin peaking at day 2 and also produced the highest amount of biomass (9.94 mg/ml). The sharp peak on day one in the SCFM+C cultures was caused by the precipitated casein in the growth medium although the biomass was washed three times using Milli-Q water before freeze drying. SCFM without supplement was the least supportive of growth.

All cultures experienced a decrease in the medium pH at the beginning of culture (Fig 1C and 1D). The pH then increased to 9 in mucin and casein supplemented SCFM media and 7.5 in SCFM only. The decrease in pH was probably due to lactic acid fermentation in the presence of glucose [55]; after glucose had been exhausted, the pH started to increase due to ammonia fermentation [56]. In the SCFM medium without supplements, the decrease of pH in the culture supernatant was slight and occurred later. This might relate to the poor growth of *S*. *aurantiacum* in SCFM (Fig 1A and 1B).

To assess the total protease activity in each culture supernatant, azocasein was used as a substrate and comparison was made between the two strains and culture time. The clinical and environmental strains exhibited a great difference in the protease activity in each cultivation medium. The highest protease activity (4.73 μg azocasein digested per minute per mg of dry biomass) was found in the culture supernatant of the clinical strain grown in the mucin supplemented medium (Fig 1E); this was over two times higher than the activity produced by the environmental strain (Fig 1F), supporting the hypothesis that the clinical strain was thriving in the presence of mucin, which is abundant in the CF lungs [57] by growing well and producing proteases (Fig 1A and 1E). The dominant form of mucin in the porcine mucin used in this study is similar to the human mucin [58, 59].

**Fig 2:**
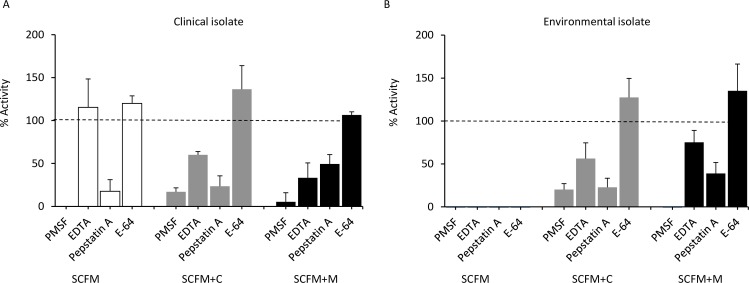
Inhibition of protease activity in day-4 culture supernatant of *S*. *aurantiacum* in three media. Percentage changes in the protease activity in the supernatant with an inhibitor relative to the non-inhibited control supernatant, set as 100% for each culture (dotted line). Data represent mean ± SD (3 biological repeats). A. Clinical strain; B. Environmental strain. SCFM: Synthetic CF sputum medium; SCFM+C (casein added); SCFM+M (mucin added).

Protease inhibitors were used to inhibit specific classes of proteases secreted by *S*. *aurantiacum* in the different day-4 culture media in order to explore the protease profile. The inhibitor concentrations used were chosen based on literature and previous testing in the laboratory (data not shown). The serine protease inhibitor PMSF showed the greatest inhibition in all cultures overall (Fig 2A and 2B) indicating that serine proteases were responsible for the majority of proteolytic activity. Pepstatin A (aspartic protease inhibitor) was the second strongest inhibitor followed by EDTA (metalloprotease inhibitor) across all media, suggesting secretion of metallo and aspartic proteases by both *S*. *aurantiacum* WM 06.482 (clinical isolate) and WM 10.136 (environmental isolate; Fig 2A and 2B). E-64 (cysteine protease inhibitor) had no effect on protease activity in any of the cultures, suggesting that *S*. *aurantiacum* did not secrete cysteine proteases under the culture conditions used. The ‘zero activity’ in the supernatants of the environmental strain grown on SCFM may reflect the fact that the overall protease activity was low to start with (Fig 1F; Fig 2B).

### Protease assays using specific substrates

**Fig 3:**
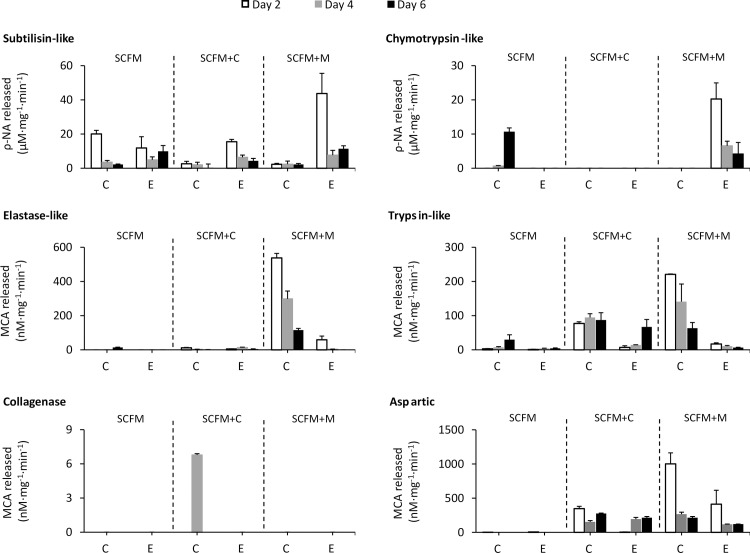
Classes and activities of specific secreted proteases in three media. Activities of specific proteases were expressed as the amount of ρ-NA or MCA released in one minute per mg of dry biomass. Substrates and test conditions are given in Table 1. Data represent mean ± SD (3 biological repeats). SCFM: Synthetic CF sputum medium; SCFM+C (casein added); SCFM+M (mucin added). C: Clinical strain, E: environmental strain.

Specific sbstrates were then used to measure the activity of each type of protease secreted by the two *S*. *aurantiacum* strains cultured in different media at their optimal pH (Table 1). Samples taken at days 2, 4 and 6 were examined. Both strains were found to secrete different proteases in response to different supplements (casein or mucin) added in the SCFM medium. Subtilisin-like serine protease activity was found to be common in all cultures although the amount varied greatly (Fig 3). Trypsin-like protease activity was also found across the cultures but it was almost close to zero in the SCFM medium. Strong presence of serine proteases is in accordance with the inhibitor studies (Fig 2).

The highest subtilisin-like activity was measured on day 2 in the environmental strain grown on the mucin supplemented SCFM medium (Fig 3). The highest trypsin-like activity as well as the highest elastase-like and aspartic protease activity was recorded for the clinical isolate on day 2 on mucin-supplemented medium, (Fig 3). The highest chymotrypsin-like activity was detected in the environemental strain on day 2 in the mucin supplemented SCFM whereas chymotrypsin activity produced by the clinical strain was very low under the same circumstances. Cysteine protease activity was not detected in any of the cultures (data not shown). Overall, the casein supplemented SCFM medium (SCFM+C) did not induce production of proteases very well except for collagenase activity in the clinical strain measured on day 4 (Fig 3).

The mucin-containing SCFM medium (SCFM+M) supported production of a broad range of proteases and the activity of each class was also, in most cases, higher compared to the other two media. Subtilisin-like, chymotrypsin-like, elastase-like, trypsin-like serine proteases and aspartic proteases were all detected. Elastase-like, trypsin-like and aspartic protease activities of the clinical strain WM 06.482 were significantly higher (about 2.5–75 fold, p < 0.05) than those of the environmental strain WM 10.136 (Fig 3). In particular, the elastase-like protease activity in the clinical isolate was high in the mucin-supplemented medium but almost undetectable in all other cultures.

To conclude, the nature of the growth medium (SCFM supplemented with casein or mucin) affected the levels and types of proteases secreted into the cultivation medium by the *S*. *aurantiacum* strains. Of particular interest in the context of proteases being described as a virulence factor, higher protease activity in the mucin supplemented SCFM provided an indication towards this role as the medium mimics the lung environment of CF patients. In this medium, serine proteases formed up 95% activity of the secreted proteases in the clinical isolate (Fig 2). Furthermore, amongst serine proteases, elastase-like and trypsin-like activities were significantly higher in the clinical isolate compared to the environmental isolate (Fig 3, p < 0.05). These two protease-types warrant further investigation as the major contributors to secreted protease activity by the clinical strain.

### Enzyme activity zymograms

**Fig 4:**
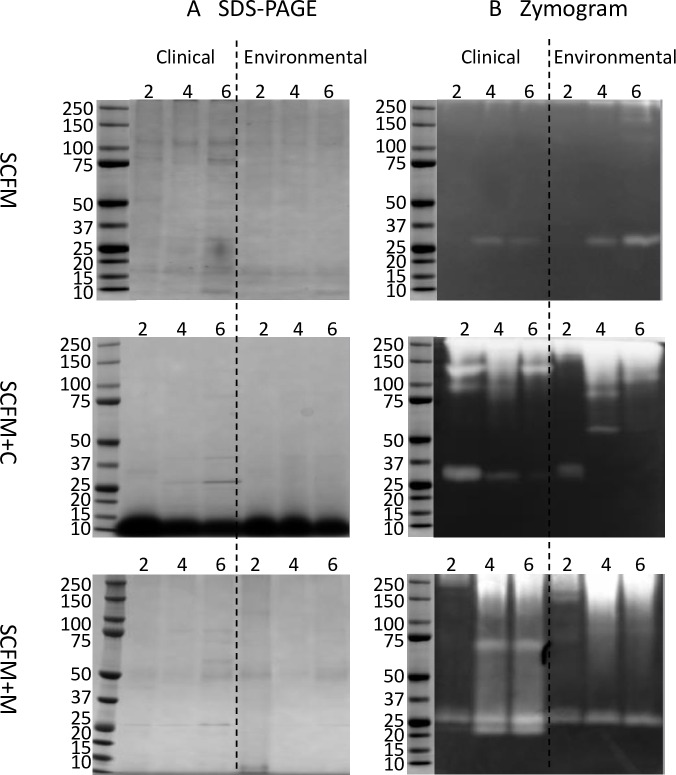
SDS-PAGE and zymogram analysis of *S*. *aurantiacum* culture supernatants. A. Proteins from the non-concentrated culture supernatant separated by protein electrophoresis. B. Enzyme activity zymograms containing 0.1% (w/v) casein as substrate. For protein gels without substrate, the supernatant was heated at 70°C for 10 min and the electrophoresis was run at room temperature. For the zymogram analysis, the supernatant was not heated, the electrophoresis was run at 4°C and incubation was conducted in Tris-HCl buffer pH 7.5 at 37°C for 16 hours. All gels were stained by Coomassie Brilliant Blue G-250. SCFM: Synthetic CF sputum medium; SCFM+C (casein added); SCFM+M (mucin added). d2, d4, d6: culture days.

The samples from day 2, day 4 and day 6 culture supernatants of the clinical (WM 06.482) and environmental (WM 10.136) isolates were further analyzed by protein electrophoresis and zymogram gels containing 0.1% (w/v) casein as substrate. The pH of zymogram gels was adjusted to 7.5 for visualization of the protease activity as this is close to the pH of the upper and lower airways of the human lungs in both CF and non-CF subjects [60]. In spite of the fact that only faint protein bands showed up on the protein gels from the culture supernatants due to the low concentration of secreted proteins (Fig 4A), clear proteolytic bands could be seen on the zymograms (Fig 4B). A proteolytic band with a molecular weight (MW) of approximately 30 kDa was common for most of the cultures, appearing in the three media at different time points in both isolates (Fig 4B).

In addition to this 30 kDa band, the clinical isolate cultured in SCFM plus casein (SFCM+C) displayed two proteolytic bands with molecular weights of approximately 100 kDa and 135 kDa in the day 2 supernatant, and also a 135 kDa band in days 4 and 6. In terms of the environmental isolate cultured in casein supplemented SCFM, two proteolytic bands with molecular weights of about 60 kDa and 80 kDa were visible on the zymogram gels on day 4 (Fig 4B). Apart from these well separated bands, all culture supernatants obtained from the casein-supplemented medium displayed a diffuse digested region of 150–250 kDa (Fig 4B).

Zymogram gels of proteins from the mucin-supplemented SCFM (SCFM+M; Fig 4) showed that in addition to the bands common for both strains, the clinical strain produced two additional proteolytic bands with molecular weights of 22 kDa and 70 kDa respectively (Fig 4B). Also, both isolates exhibited a digested area of about 150–250 kDa at the top of the zymogram as well as a distinct proteolytic band of about 30 kDa in all cultures (Fig 4B).

Drawing a parallel to the protease diversity assays (Fig 3), subtilisin-like and trypsin-like activities were detected in all cultures using specific substrates. As subtilisins typically have molecular weights of about 20–45 kDa [61], and some fungal trypsin-like proteases also have a similar molecular size [62, 63], the 30 kDa protein detected in the zymograms could belong to either of these two groups of proteases. However, as many elastases also fall within this size range [64], it is difficult to draw more detailed conclusions from the zymogram gels only. In the mucin-supplemented medium, the two proteolytic bands of about 70 kDa and 22 kDa were produced by the clinical isolate only (Fig 4B). It could be speculated that these two proteolytic bands were produced by elastase-like, trypsin-like or aspartic proteases (Fig 3), secreted by the clinical isolate. Verification of these deliberations was attempted by MS/MS analysis (see below).

### Identification of secreted proteases by mass spectrometry

**Fig 5:**
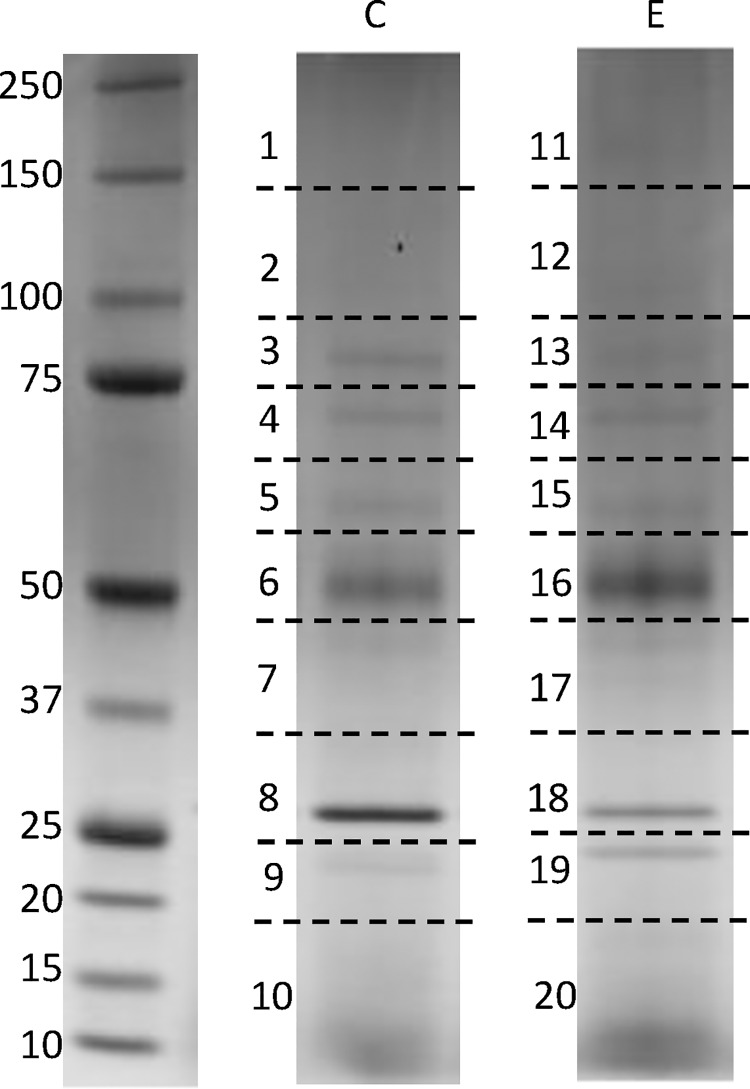
SDS-PAGE of proteins concentrated from the *S*. *aurantiacum* culture supernatants. 20 μg of protein was loaded in each well. The gel was cut horizontally into 10 pieces and the sections were numbered as shown. Note that some sections did not contain visible protein bands. Peptides from each single section were analysed by MS. Numbering of the bands corresponds to the numbers in Table 2. C: clinical strain, E: environmental strain.

After the broad profiling of protease activity, further studies focused on the secreted proteases produced by the clinical isolate WM 06.482 and the environmental isolate WM 10.136 in the mucin supplemented SCFM as more proteases were secreted by the clinical isolate in this medium and the composition of the medium mimics that of the lung fluid. Proteins of the day 4 culture supernatants were concentrated and separated on SDS-PAGE and each lane on the gel was cut horizontally into 10 pieces, as shown in Fig 5. The protein in each piece was digested by trypsin and GluC separately, in three biological triplicates. The extracted peptides were analyzed by LC-MS/MS and data were searched against a manually assembled database consisting of all available fungal protease sequences in NCBI, as only a non-annotated draft genome is available for *S*. *aurantiacum* at the moment [65, 66]. The search results from both trypsin and GluC digestion were combined together.

Identification of a protein was considered valid only when the protein was present in all three biological replicates and the total number of matched peptides was more than five. Six homologs of fungal proteases (Table 2) were identified from the clinical isolate and five from the environmental isolate. Three protease homologs were common for both isolates including a subtilisin protease S8, a putative leucine aminopeptidase and a PA-SaNapH-like protease. The NCBI data showed that these proteases were also present in *S*. *apiospermum*, which is placed in the same genus as *S*. *aurantiacum* [67].

**Table 2:** Proteases identified by LC-MS/MS from day-4 culture supernatants of *S*. *aurantiacum* WM 06.482 (clinical isolate) and WM 10.136 (environmental isolate) grown in mucin supplemented SCFM. Full lists of identified proteins are given in supporting information S1 and S2 Tables.

Identified proteins	NCBI accession No.	Theoretical pI	Theoretical MW (kDa)	Score /log (e)	Homologous to	Protein matches	
						Section No.	No. of Peptides found
**Clinical strain**							
Peptidases S8, subtilisin family	gi|666867549|	8.2	41.2	-43.6	*Scedosporium apiospermum*	8	226
Putative leucine aminopeptidase, M28 Zn-peptidase	gi|666869274|	5.22	53.7	-28.9	*Scedosporium apiospermum*	5	15
PA SaNapH like protease	gi|666869012|	4.84	52.7	-10.1	*Scedosporium apiospermum*	6	14
Peptidase C48	gi|114192178|	10.46	100.3	-2.3	*Aspergillus terreus*	10	7
Pepsin-like aspartate, Asp	gi|799243341|	5.66	51.8	-2.2	*Hirsutella minnesotensis*	3	6
Trypsin-like serine protease	gi|573992599|	4.46	32.7	-2.3	*Cordyceps militaris* CM01	9	7
**Environmental strain**							
Peptidases S8, subtilisin family	gi|666867549|	8.2	41.2	-51.9	*Scedosporium apiospermum*	18	94
Putative leucine aminopeptidase, M28 Zn-peptidase	gi|666869274|	5.22	53.7	-15.1	*Scedosporium apiospermum*	15	15
PA SaNapH like protease	gi|666869012|	4.84	52.7	-11.3	*Scedosporium apiospermum*	16	17
Pepsin-like aspartic protease	gi|639569211|	5.75	103.4	-2.9	*Tilletiaria anomala*	14	7
KLLA0E06711p	gi|49643385|	6.94	83.1	-3.8	*Kluyveromyces lactis*	14	19

The number of peptides found represents the sum of peptides found in the three biological replicates. Log (e) value was calculated as the base-10 log of the expectation that any particular protein assignment was made at random (mean of three biological replicates). The SDS-PAGE gel with the numbered cut-out sections is shown in Fig 5.

The homolog of subtilisin protease identified by MS from the gel fractions numbered 8 and 18 (Fig 5), corresponds to the proteolytic band of around 30 kDa on the zymogram gel which showed up in all cultures (Fig 4B). The fact that subtilisin-like protease activity was detected in all cultures with some difference between the clinical and environmental isolates (Fig 3) is consistent with the MS and zymogram analyses.

Homologs of putative leucine aminopeptidase and PA-SaNapH-like protease (Table 2) were identified by mass spectrometry from both strains. However, their corresponding proteolytic bands were not detected in the zymograms (Fig 4B), indicating that these proteases were not capable of proteolysis of casein in the zymogram gel or had lower specific activities compared to subtilisin since the proteolytic band of subtilisin was visible on the zymogram gels. These two protease homologs were identified from the sections of 5 and 6 (15 and 16 for environmental strain, Fig 5) respectively, and the ranges of their MW were similar to the MW of homologous proteins in *S*. *apiospermum* (Table 2).

A homolog of trypsin-like serine protease was identified from the cultures of the clinical isolate WM 06.482 (Fig 5, section 9), matching the 22 kDa proteolytic band on the zymogram gel (Fig 4B), displayed only in the clinical cultures. Enzyme activity assays also demonstrated that the clinical isolate secreted higher amounts of trypsin-like activity than the environmental isolate (Fig 3); correspondingly, the proteolytic bands of around 22 kDa present only in the zymogram of the clinical s could be assumed to belong to potentially represent a trypsin-like protease (Fig 4B) despite the fact that the day 2 culture supernatant that showed higher trypsin-like protease activity than the day 4–6 samples (Fig 3), produced only a faint proteolytic band (Fig 4B).

In addition to homologs of a fungal subtilisin peptidase, putative leucine aminopeptidase, PA-SaNapH-like protease and a trypsin protease, the clinical isolate was found to secrete a peptidase C48 homolog (gi114192178), and a pepsin-like aspartate protease homolog (gi799243341). These proteases did not produce distinct bands on the zymogram gel (Fig 4), indicating that they were not able to degrade casein in the zymogram or their specific activity were quite low. The peptidase C48 was identified from section 10 with a MW < 20 kDa which was quite different from MW of the homologous *A*. *terreus* protein (100.3 kDa, Table 2). It may be that peptidase C48 has been degraded by other coexisting proteases in the culture supernatant, or because the peptidase C48 homolog identified from *S*. *aurantiacum* was only matching to one of the domains of the reference protein.

## Discussion

We have studied a clinical isolate *S*. *aurantiacum* WM 06.428 and an environmental isolate WM 10.136 with a view of establishing the profile of proteases secreted by these strains and detecting potential differences in protease production related to their lifestyle.

Similarly to the studies carried out with the opportunistic fungal pathogen *Aspergillus* [68, 69], the amount and type of proteases secreted by *S*. *aurantiacum* varied according to the medium composition (Fig 1E and 1F, Fig 3). Among all cultures, the highest overall protease activity was detected in the culture supernatant of the clinical isolate grown in mucin-supplemented SFCM (SCFM+M). Differently to the clinical isolate, the environmental strain produced the highest general protease activity in the casein-supplemented medium (SCFM+C). Elastase activity was primarily found in the culture supernatants of the clinical isolate whereas KLLA0E06711p, a protease homologous to a *Kluyveromyces lactis* protease showing metalloendopeptidase activity (http://www.uniprot.org/uniprot/Q6CP89), was only identified in the culture supernatant of the environmental strain.

High mucin concentration is one of features of CF, which makes the mucous lining of some organs thick and sticky favoring growth of microorganisms [70]. Accordingly, the clinical *S*. *aurantiacum* isolate produced over 2 fold higher total proteolytic activity in responding to the excessive mucin when compared to the environmental isolate (Fig 1E and 1F). This ability also explained why the growth rate of the clinical strain was found to be the greatest on the mucin-supplemented SCFM medium where the growth also peaked earlier than in other cultures (Fig 1A and 1B). It has been suggested that serine proteases, elastase-like in particular, are responsible for mucin digestion in the cystic fibrosis lungs [71]. In our study, elastase-like activity was mainly detected in the culture supernatant of the clinical isolate (Fig 3), which may also contribute to mucin degradation [71]. Indeed, serine proteases were the dominant protease class secreted by the clinical strain (Fig 2). Our study further demonstrated that the elastase-like and trypsin-like serine proteases as well as aspartic proteases displayed 2.5–75 fold higher activities in the clinical isolate compared to the environmental isolate (Fig 3).

Studies on elastase secreted by *A*. *fumigatus* showed that this enzyme could be inhibited by both PMSF (serine protease inhibitor, 100% inhibition) and EDTA (metalloprotease inhibitor 100% inhibition) [64]. These two compounds also showed the strongest inhibitory effect on the proteases produced by clinical isolate *S*. *aurantiacum* WM 06.482 (Fig 2). This points to the possibility that the *S*. *aurantiacum* protein exhibiting elastase activity may have similar properties to the *A*. *fumigatus* enzyme. Since some proteases, such as elastase, can be inhibited by multiple classes of inhibitors [64], the percentage values of inhibition cannot be simply added together (Fig 2).

The elastase-like activity produced by *S*. *aurantiacum* in the mucin medium, detected in class-specific substrate studies was not picked up by LC-MS/MS, although some peptides identified from section 7 of the protein gel (Fig 5) aligned with elastinolytic zinc metalloprotease (LasB) identified from various fungi. However, the identity score was very low and less than five peptides showed a match (S1 Table). On the other hand, the peptides displayed above aligned with the zinc metalloprotease (elastase) LasB (gi|398055407) of *Brevibacillus sp*. CF112 very well (S1 Table). This may indicate that either the amino acid sequence of *S*. *aurantiacum* elastase is different to other fungi but more similar to that from some bacteria or the elastase-like protease was of low abundance but had particularly high activity against the specific substrate N-Sue-Ala-Ala-Ala-MCA.

A trypsin-like serine protease homolog was also identified by mass spectrometry (Table 2) from the culture supernatant of the clinical strain grown on mucin-supplemented medium. This 22 kDa enzyme generated a clear proteolytic band on the zymogram gel (Fig 4B). The finding is in accordance with the enzyme activity assays displayed in Fig 3. Fungal extracellular trypsin-like proteases may participate in the pathogenic process since a correlation between trypsin-like activity and fungal pathogenicity has been identified and almost all fungi containing genes encoding trypsin-like proteases are pathogens of plants, animals or other fungi [72–74]. Thereby the trypsin-like enzyme(s) produced by *S*. *aurantiacum* also deserve a further study.

Mass spectrometry revealed a number of proteases that were not detected in the current study by enzyme activity assays. This could be because of the range of selected substrates and focusing on more common fungal proteases. On the other hand, the trypsin like protease discussed above was only identified from the clinical strain by mass spectrometry even though trypsin-like activity was detected in both strains. This may be because of low abundance of the protein in the culture supernatant of the environmental strain. Availability of an annotated genome sequence of *S*. *aurantiacum* would help in resolving these questions in the future.

## Conclusions

The clinical and environmental *S*. *aurantiacum* isolates differ in their capacity to produce secreted proteases. This was reflected both by the response of the two strains to the different culture media and the amount of a particular type of protease produced. The proteases were dominated by serine proteases, and activities of elastase-like and trypsin-like proteases produced by the clinical isolate on mucin-supplemented SFCM were up 2.5–75 fold compared to those produced by the environmental strain. There are indications that the elastase-like protease of *S*. *aurantiacum* might be different to other fungal elastases reported in the NCBI database or this protease was of low abundance but its activity was particularly high. To our knowledge, this is the first study on proteases secreted by *S*. *aurantiacum*.

## Supporting Information

S1 Table Identification data of the proteins from clinical isolate WM 06.482 listed in Table 2. (XLSX)

S2 Table Identification data of the proteins from environmental strain WM 10.136 listed in Table 2. (XLSX)
